# Genomic Instability Profiles at the Single Cell Level in Mouse Colorectal Cancers of Defined Genotypes

**DOI:** 10.3390/cancers13061267

**Published:** 2021-03-12

**Authors:** Vasilis S. Dionellis, Maxim Norkin, Angeliki Karamichali, Giacomo G. Rossetti, Joerg Huelsken, Paloma Ordonez-Moran, Thanos D. Halazonetis

**Affiliations:** 1 Department of Molecular Biology, University of Geneva, 1211 Geneva, Switzerland; vasileios-stamatios.dionellis@unige.ch (V.S.D.); angeliki.karamichali@unige.ch (A.K.); giacomo.rossetti@unige.ch (G.G.R.); 2 Cancer Stem Cell Laboratory, Swiss Institute of Technology Lausanne (EPFL), ISREC, 1015 Lausanne, Switzerland; maxim.norkin@epfl.ch; 3 Division of Cancer & Stem Cells, School of Medicine, Centre for Cancer Sciences, Biodiscovery Institute, University of Nottingham, Nottingham NG7 2RD, UK

**Keywords:** colorectal cancer, single cell, genomic instability, exome sequencing, mouse models, cancer drivers, single nucleotide variants, copy number alterations, mutational signature, organoids

## Abstract

**Simple Summary:**

Colorectal cancer (CRC) is one of the leading causes of cancer mortality; it typically originates as adenomas that progress over time to carcinomas. We decided to investigate the accumulation of numerous genomic alterations during tumour progression by using a mouse model with three different targetable alleles that can be found in human colorectal cancers. We conclude that the rate of accumulation of SNSs is higher in transformed compared to non-transformed cells, and that it is unaffected by the number of cancer-driver genes that are active in the tumour.

**Abstract:**

The genomes of many human CRCs have been sequenced, revealing a large number of genetic alterations. However, the molecular mechanisms underlying the accumulation of these alterations are still being debated. In this study, we examined colorectal tumours that developed in mice with *Apc^lox/lox^*, *LSL-Kras^G12D^*, and *Tp53^lox/lox^* targetable alleles. Organoids were derived from single cells and the spectrum of mutations was determined by exome sequencing. The number of single nucleotide substitutions (SNSs) correlated with the age of the tumour, but was unaffected by the number of targeted cancer-driver genes. Thus, tumours that expressed mutant *Apc*, *Kras,* and *Tp53* alleles had as many SNSs as tumours that expressed only mutant *Apc*. In contrast, the presence of large-scale (>10 Mb) copy number alterations (CNAs) correlated strongly with *Tp53* inactivation. Comparison of the SNSs and CNAs present in organoids derived from the same tumour revealed intratumoural heterogeneity consistent with genomic lesions accumulating at significantly higher rates in tumour cells compared to normal cells. The rate of acquisition of SNSs increased from the early stages of cancer development, whereas large-scale CNAs accumulated later, after *Tp53* inactivation. Thus, a significant fraction of the genomic instability present in cancer cells cannot be explained by aging processes occurring in normal cells before oncogenic transformation.

## 1. Introduction

The colon is an excellent organ for studying the accumulation of genomic alterations during cancer development because precancerous and cancerous lesions can be easily harvested [1,2,3,4,5,6]. The initial event in most colorectal carcinomas (CRCs) is biallelic inactivation of the tumour-suppressor gene *Adenomatous polyposis coli* (*APC*) [2,7,8,9]. Further CRC development involves the accumulation of additional mutations that may be subclone-specific [6,10,11,12,13,14,15]. Activating mutations targeting *KRAS* are acquired in up to 40–50% of sporadic CRCs and are associated with dysplasia [2,8,16]. Up to 50–60% of human CRCs acquire inactivating mutations in the *TP53* tumour-suppressor gene, an event associated with progression of dysplastic lesions to carcinoma. p53, the protein product of *TP53*, induces cell cycle arrest, senescence, or apoptosis in response to DNA damage. Thus, its inactivation allows cancer cells to survive and proliferate despite the presence of oncogene-induced DNA damage [17].

Identification of the genes that are frequently mutated in human CRC allowed mouse models to be generated. The first model to be generated harboured a deletion in one allele of the *Apc* gene (*Apc^min/+^*) [18]. Although this model has been studied extensively, it does not fully recapitulate human CRC because the tumours develop mainly in the small intestine and they are benign adenomas [18,19]. Another mouse model has mutations in both the *Apc* and *Kras* genes; these mice show higher tumour multiplicity than *Apc^min^* mice and more importantly the colonic tumours invade the intestinal mucosa [16,20,21]. As *TP53* inactivating mutations are frequent in advanced human CRC, yet another mouse model was generated by combining mutations in the *Apc*, *Kras* and *Tp53* genes (AKP model). In these mice, aggressive carcinomas develop in the ceacum and colon [22,23]. Moreover, cell lines established from these tumours are able to metastasise to the liver after intrasplenic injection or orthotopic transplantation into immunodeficient mice [22,24].

One of the most important hallmarks of cancer, including CRC, is genomic instability, a feature that facilitates cancer progression [25] and resistance to therapy [11,26,27]. Genomic instability can lead to the accumulation of numerous genomic alterations, including single nucleotide substitutions (SNSs), small insertions and deletions (indels), copy number alterations (CNAs), and chromosomal rearrangements. It is well established that CNAs and chromosomal rearrangements accumulate at higher rates in cancer cells than in normal cells. However, it is less clear whether the rate of acquisition of SNSs increases after cell transformation. The early consensus in the field has been that the high number of SNSs in most human cancers simply reflect the high number of point mutations present in normal cells due to aging; since tumours are of monoclonal origin, these mutations become evident when tumour DNA is sequenced [28]. An alternative view is that SNSs accumulate at higher rates in cancer cells. Our sequencing study of human colon adenomas supported this latter view, since it revealed a higher number of SNSs in adenomas with severe dysplasia, compared to adenomas with mild dysplasia, despite similar patient age distribution [29]. One may also consider the possibility that certain types of mutations accumulate at higher rates in cancer cells, whereas other types of mutations accumulate at equal rates in normal and cancer cells due to, for example, aging. Along these lines, it is worth noting that the large-scale sequencing studies of human cancers have revealed distinct types of SNSs that are referred to as mutational signatures [30,31,32,33]. Various bulk tissue sequencing studies of genetically engineered mouse models (GEMMs), that recapitulate aspects of human cancers have also revealed a spectrum of SNSs [33,34,35,36,37,38,39,40].

The prevailing signature in human cancers is signature 1, a signature that is defined by a high number of C-to-T transitions in the context of CpG sites [32,41]. These mutations arise from failure to properly repair a methylated cytosine, after it has been deaminated by hydrolysis [42]. It has been proposed that signature 1 mutations accumulate with equal rates in normal and cancer cells [43,44,45]. However, the majority of mutations in colon cancer conform to signature 1 and our exome sequencing study, cited above, revealed a higher number of signature 1 SNSs in adenomas with severe dysplasia, compared to mild dysplasia. A higher mutation rate for signature 1 SNSs can be rationalised on the basis that cancer cells have DNA replication stress, which leads to the formation of single-stranded DNA [17]. The rate of cytosine deamination is a hundred times higher in single-stranded than in double-stranded DNA [42], and deamination of a methylated cytosine to thymine in single-stranded DNA cannot be detected by the repair machinery because it does not lead to base pair mismatches and because thymine is a naturally occurring base in DNA.

Understandably, interpretation of cancer sequencing data is complicated by the presence of intratumoural heterogeneity [28,46]. Mutations that are present in a subset of cancer cells may have very low overall allele frequencies and not be counted. To address this problem, one can prepare single cell-derived tumour organoids for sequencing. In a previous study, we sequenced the exomes of organoids derived from normal or precancerous single cells isolated from the intestines of *Apc^min/+^* mice and observed eleven times more mutations in the organoids derived from the precancerous cells [47]. Another study also examined single cell-derived organoids; in this latter study, organoids were prepared from cancer and normal cells obtained from three CRC human patients. Comparison of the number of mutations in the tumour-derived organoids, as compared to the normal tissue-derived organoids, revealed a modest increase in the number of SNSs conforming to signature 1 and a more significant increase in the number of SNSs conforming to signature 17 [48].

To gain a better understanding of mutation rates in cancer cells, we turned to a mouse model of CRC that is driven by three cancer driver genes. Specifically, we examined mice that had three targetable alleles: *Apc^lox/lox^*, *LSL-Kras^G12D^*, and *Tp53^lox/lox^*, corresponding to the most frequently mutated genes in human colon cancer [49,50]. The mice also harboured a transgene that was expressed specifically in the colon and which encoded a tamoxifen-inducible recombinase (*Cdx2^CreERT2^*), allowing the three cancer-driver genes to be targeted in an inducible manner. As before, we prepared single cell-derived organoids from CRCs that developed in these mice and sequenced their exomes. Our results provide a better understanding of the role of oncogenes and tumour suppressor genes on the accumulation of SNSs and CNAs in cancer cells.

## 2. Results

### 2.1. Clonal Organoid Cultures Derived from Single Tumoral Cells

**Figure 1:**
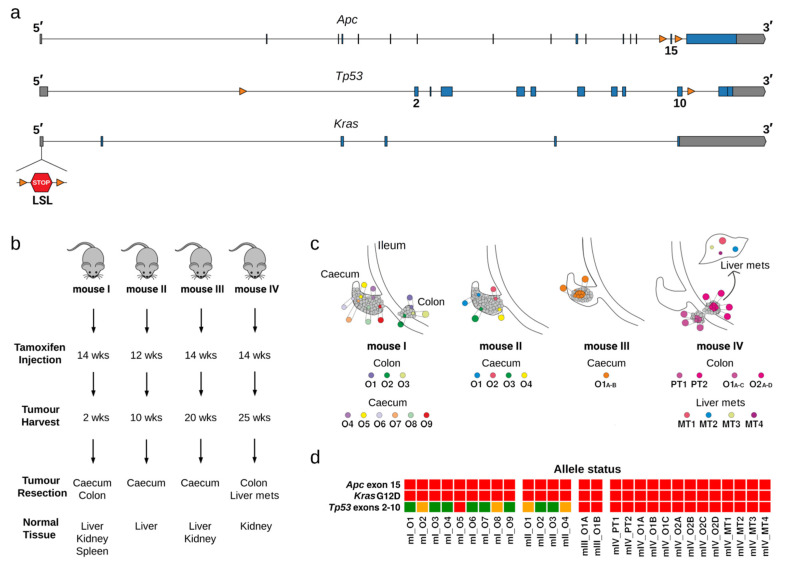
Colorectal carcinoma (CRC) mouse model, resected samples, and genotype of organoids. (**a**) Structure of the conditional alleles *Apc^lox/lox^*, *Tp53^lox/lox^*, and *LSL-Kras^G12D^*. Blue boxes and orange triangles indicate exons and loxP sites, respectively. (**b**) Four 3–4-month-old mice were injected with tamoxifen to initiate tumour development. Caecum and colon tumours were obtained for subsequent isolation of intestinal single cells. Normal tissue was also resected and used as control sample during sequencing analysis. Mouse IV developed metastatic lesions in the liver and metastatic tumour samples were also resected and sequenced. (**c**) Multi-region sampling of each mouse is illustrated by coloured labels. PT: primary tumours, MT: metastasis, O: tumour organoids. (**d**) Recombination events in organoids validated by PCR-based genotyping and by read depth analysis of sequencing data. Red, orange, and green colour indicate homozygous, heterozygous and no recombination for *Apc^lox/lox^* and *Tp53^lox/lox^*, respectively. For *LSL-Kras^G12D^*, red colour represents recombination of the conditional allele.

We used a mouse model of colonic tumorigenesis (AKP-*Cdx2^CreERT2^* mice), in which three endogenous cancer-driver genes were modified, so they could be targeted by a tamoxifen-inducible *Cre* gene. The targeted cancer-driver genes were *Apc*, whose exon 15 was flanked by loxP sites (*Apc^lox/lox^*), *Kras*, which contained a G12D mutation and a transcription termination site flanked by loxP sites upstream of the first coding exon (*Kras*^LSL-G12D/+^), and *Tp53*, whose exons 2–10 were flanked by loxP sites (*Tp53^lox/lox^*) (Figure 1a and Figure S1a). The *Cre* gene was under control of the *Cdx2* promoter to confer specific expression in the large intestine.

We administered tamoxifen to four mice (Figure 1b). The first mouse (mI) received a dose of 30 mg/kg, which led to multiple transformation events across the whole caecum; this mouse was sacrificed two weeks after tamoxifen administration. The other three mice received a low tamoxifen dose of 3 mg/kg, which led to low levels of recombination and few tumours developing. Mouse II was sacrificed ten weeks after tamoxifen injection. Mice III and IV were sacrificed 20 and 25 weeks after tamoxifen injection, respectively. These mice developed carcinomas, which invaded the bowel wall and reached the peritoneum (Figure S1c). Tissue biopsies with macroscopically visible tumours were used to prepare suspensions of single cells, which were then aliquoted into wells of 96-well plates for expansion as 3D-organoid cultures (Figure 1c and Figure S1b). We only propagated cultures from wells, in which initially only a single organoid grew, and we considered that these cultures were derived from a single cell. The organoids were spheroid-shaped and lacked the crypt-like projections that are typical of intestinal organoids derived from non-transformed cells (Figure S1b).

A diagnostic PCR, supported by analysis of the number of exome sequencing reads, was used to determine whether the *Apc*, *Kras^G12D^*, and *Tp53* genes had undergone recombination by Cre (Figure 1d). As expected, all tumour-derived organoids retained the *Cre* gene (Figures S2a and S11) and had suffered biallelic deletions of exon 15 of *Apc* (Figures S2b, S3 and S11). The *Kras^G12D^* allele had also recombined in all organoids (Figure 1d; Figures S2c, S4, S5 and S11). Finally, in most organoids derived from mice I and II, the *Tp53* gene had not recombined, whereas in all organoids from mice III and IV both *Tp53* alleles had recombined (Figure 1d; Figures S2d, S6 and S11).

### 2.2. Single Nucleotide Substitutions—Prevalence

Single nucleotide substitutions (SNSs) were identified by comparing the exome sequencing data of single cell-derived tumour organoids to the corresponding data of bulk normal tissue from the same mouse (Figure 1b). We used the liver as the reference normal tissue, except for mouse IV, for which we used the kidney, because its liver had metastatic lesions (Figure 1c and Figure S1c). For mouse IV, we also obtained exome sequencing data of primary and metastatic tumour tissue biopsies (Figure 1c and Figure S1c).

One concern when analysing cancer sequencing data, in which few SNSs are expected per sample, is that a significant fraction of the identified variants may be germline polymorphisms and not somatic SNSs. This type of error will occur, when, due to low sequencing coverage, germline variants are identified in the sequences of the tumour organoid, but not in the sequences of the reference normal tissue. Given the very large number of non-annotated germline variants in the mouse, such errors may be frequent. To minimise them, we performed exome sequencing, which allowed us to have high sequencing coverage. In addition, we restricted the analysis to the protein-coding regions, because these are well-annotated.

**Figure 2:**
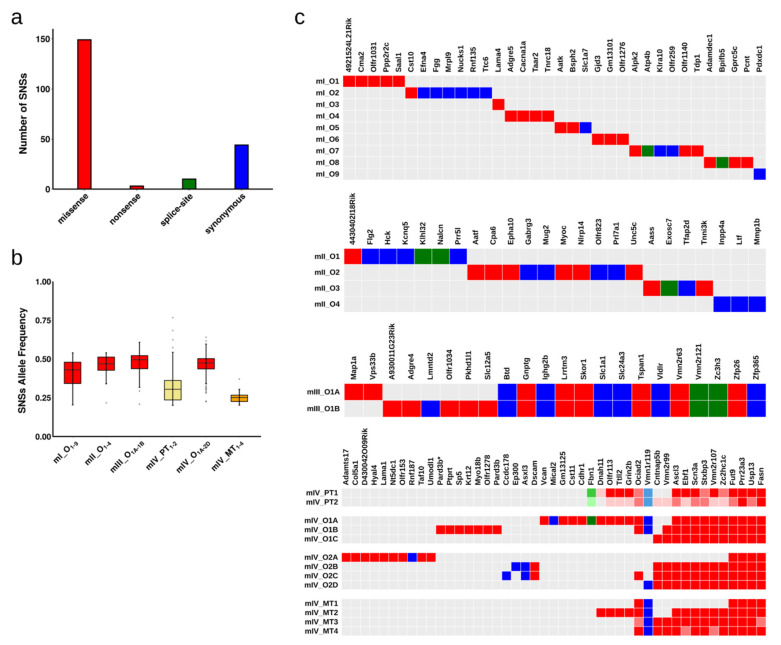
Types and allele frequencies of identified point mutations. (**a**) Number of single nucleotide substitutions (SNSs) within the protein-coding regions that were identified in the tumour organoids of all four mice. (**b**) Allele frequencies of coding and splicing somatic SNSs present in tumour organoids of mice I-IV and in primary and metastatic tumours of mouse IV. As expected, the average allele frequencies of the SNSs in the organoids were approximately 50% (red boxes), whereas in the primary and metastatic tumours (yellow and orange boxes, respectively) the SNS allele frequencies are lower. (**c**) Coding and splice site SNSs present in sequenced tissues and organoids. Red, blue, and green colours indicate nonsynonymous, synonymous and splice-site point mutations, respectively. For the primary and metastatic biopsies of mouse IV, SNSs with allele frequencies less than 20% or less than 10% are marked by boxes with decreased colour saturation.

The exome sequencing data of 22 tumour organoids prepared from the four mice revealed, in total, 206 somatic SNSs mapping to gene coding regions or splice sites (Data S1). Of the 206 SNSs, 149 were missense, 3 nonsense, 44 synonymous and 10 were targeting splice-sites (Figure 2a). We classified all the SNSs as passenger mutations, since none of them targeted known oncogenes or tumour suppressor genes (as defined by ICGC/TCGA).

The average allele frequency of the SNSs in the organoids was approximately 50% (Figure 2b). Considering that the organoids were derived from a single cell, these allele frequencies are consistent with heterozygous mutations acquired in vivo. If the SNSs had been acquired during tissue culture, they would not be present in all cells and would have lower allele frequencies. By comparison, the allele frequencies of the SNSs in bulk tumour tissue were lower than 50% (Figure 2b), reflecting intratumoural heterogeneity (see below) and the presence of normal cells in bulk tumour tissue.

We next studied the spectrum of the identified SNSs. For mice I and II, tumour biopsies that were a few mm apart from each other were used to prepare organoid cultures, and one organoid culture was sequenced per tissue biopsy (Figure 1c). In these mice, no SNSs were shared between the different organoids indicating that independent transformation events took place in the different biopsies (Figure 2c). Indeed, mice I and II showed signs of tumour development quite early after tamoxifen injection, consistent with the development of many tumours in parallel.

For mouse III, a single tumour tissue biopsy was used to obtain organoid cultures, two of which were subjected to exome sequencing (Figure 1c). Fourteen SNSs were shared between the two organoids, and eight SNSs were private (two and six, respectively, in the two organoids) (Figure 2c). At the very minimum, the private SNSs must have been acquired after tumour development was initiated.

For mouse IV, two tumour tissue biopsies were harvested; three and four single cell-derived organoid cultures were then sequenced from the two biopsies, respectively (Figure 1c). Interestingly, all organoids shared four SNSs, whereas six of the seven organoids shared an additional six SNSs (Figure 2c). Thus, the organoids derived from the two tumour biopsies were related to each other. Given that the average frequency for all mutant alleles was about 50% (Figure 2b), we infer that the SNSs were acquired in vivo. It is possible that the four shared SNSs might have been acquired prior to neoplastic transformation. In contrast, the private SNSs (29 in total; defined as SNSs found in only one of the seven organoids) and the semi-private SNSs (12 in total; defined as SNSs found in more than one, but not in all organoids) must have been acquired after neoplastic transformation (Figure 2c).

Mouse IV was the only mouse that developed metastases to the liver. Histopathological examination of primary tumours revealed the development of moderately differentiated invasive adenocarcinoma of the colon, accompanied by mild to median desmoplastic and mild inflammatory reaction of the stroma. The cancerous glands penetrated into the muscularis propria. The metastatic cells also presented moderately differentiated adenocarcinoma histology (Figure S1c). We attempted to establish organoids from the metastatic lesions, but were unable to do so (Figure 1c and Figure S1b). However, we sequenced the exomes of four distinct metastatic lesions (MT1–MT4) and also the exomes of the two primary tumour biopsies (PT1, PT2), from which we had successfully obtained organoid cultures (O1A-C and O2A-D). The four SNSs that were shared by all organoids were also present in the primary and metastatic tumour biopsies. The majority of the semi-private SNSs were present in at least one of the biopsies, whereas the majority of the private SNSs were absent (Figure 2c). The two primary tumour biopsies had similar, although not identical SNSs profiles, whereas three of the four metastatic lesions had distinct SNS profiles, indicating at least three independent metastasis seeding events (Figure 2c).

### 2.3. Single Nucleotide Substitutions—Link to Genotype and Distribution

**Figure 3:**
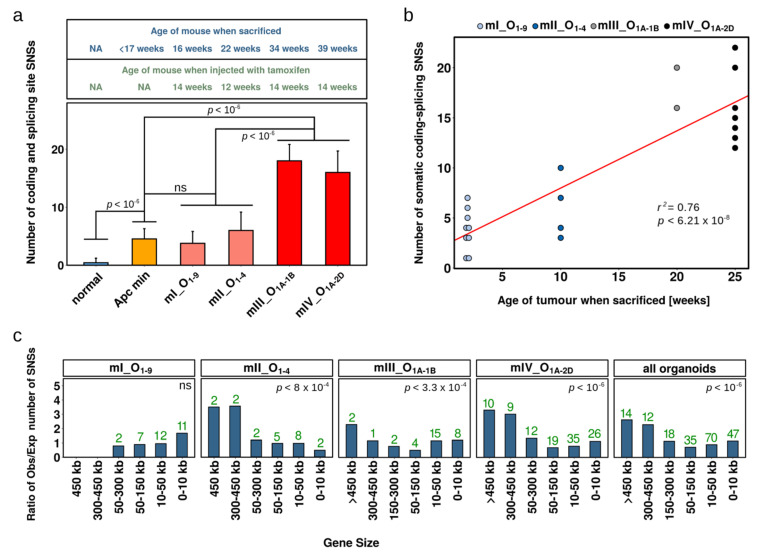
Relation of number of SNSs per organoid to age of the tumour and distribution of SNSs in the genome according to gene size. (**a**) Average number of somatic SNSs in normal and tumour organoids of *Apc^min/+^* mice and in tumour organoids of AKP mice. Organoids originating from mice I and II displayed a similar mutational burden as organoids from *Apc^min/+^* mice, whereas organoids from mice III and IV had a higher mutational burden. Data are presented as mean + 1 SD and compared using the Student’s *t* test. *p* values were corrected for multiple testing using the Benjamini–Hochberg method. (**b**) Linear regression correlation of the number of somatic coding and splice site SNSs to the time over which the tumours developed, calculated from the dates at which the mice were injected with tamoxifen and sacrificed. The *p* value and the value of the coefficient of determination R^2^ are indicated. (**c**) Distribution of SNSs according to gene size. The graphs show the ratios of observed (Obs) versus expected (Exp) number of SNSs for each gene category. The observed number of SNSs is indicated by the green letters. With the exception of mouse I, the somatic SNSs were significantly more prevalent in the large genes. Significance was evaluated by calculating the expected distribution of SNSs in the genes 0–150 kb in size versus the genes >150 kb. A random number generator was used to assign each SNS to one or the other group of genes, according to the total length of the coding sequences of each group of genes. Once all SNSs had been assigned, the number of SNSs mapping to the large genes was compared to the observed number. This process was repeated one million times, and the *p* value corresponded to the number of times that more randomly assigned SNSs mapped within large genes than what was observed.

We next examined whether there was a correlation between the genotype of the organoids and the number of SNSs acquired. The organoids from mice I and II, in which *Apc* was inactivated and mutant *Kras* was expressed, had accumulated fewer SNSs than the organoids derived from mice III and IV, in which *Tp53* was also inactivated (Figure 3a). Nevertheless, this difference might not be related to the genotype, because mice III and IV were sacrificed 20 and 25 weeks after tamoxifen administration, whereas mice I and II were sacrificed after two and ten weeks, respectively (Figure 1b). Thus, the number of SNSs in the four mice correlated well with the time over which the tumours developed (Figure 3b), implying a similar rate of SNS acquisition over time in all mice.

In a previous study, we had performed exome sequencing of single cell-derived organoids from *Apc^min/+^* mice; these organoids originated either from adenomatous polyp or normal intestine tissue. The average number of SNSs in the transformed *Apc^min/min^* organoids was very similar to the number of SNSs present in the organoids from mice I and II, which expressed mutant *Kras*, in addition to having inactivated *Apc* (Figure 3a). In contrast, the organoids derived from non-transformed cells from the same mice had significantly fewer SNSs than the transformed organoids (Figure 3a). These results suggest that expression of mutant *Kras* did not have a significant effect on the rate by which SNSs accumulate; on the other hand, transformed cells had a higher mutation rate than normal cells. We note that colon cells that have as cancer-drivers only mutant *Apc* or only mutant *Apc* and mutant *Kras* are generally precancerous in humans.

To explore whether differences in SNS acquisition rates could be explained by differences in proliferation rates, we calculated the cell doubling times in organoids with mutations in a single cancer-driver gene (*Apc*) and in organoids with mutations in three cancer-driver genes (AKP) using the formula (1) described in the Section 4.6 of Materials and Methods; the doubling times were 9.1 ± 0.4 and 8.2 ± 0.4 h, respectively (*p* = 0.08; not statistically significant). The corresponding value for the *Lgr5*+ stem cells in organoids derived from normal tissue is 12 h [22,51,52]. These values indicate that the large difference in SNSs present in non-transformed and transformed cells cannot be explained by differences in proliferation rates.

An interesting feature of SNSs in human precancerous and cancerous lesions is that they target more frequently large genes than small genes [29]. Mechanistically, one explanation is that a slower progression of replication forks in cancer cells could result in the central segments of large genes being replicated in mitosis by the break-induced replication mechanism, which is error-prone [17,53,54,55,56,57,58,59]. We examined the distribution of somatic SNSs according to gene size in the mouse tumour organoids and observed more SNSs per Mb than expected in large genes (Figure 3c). This effect was statistically significant for mice II, III and IV, but not for mouse I, which developed tumours rapidly.

### 2.4. Single Nucleotide Substitutions—Mutational Signature

**Figure 4:**
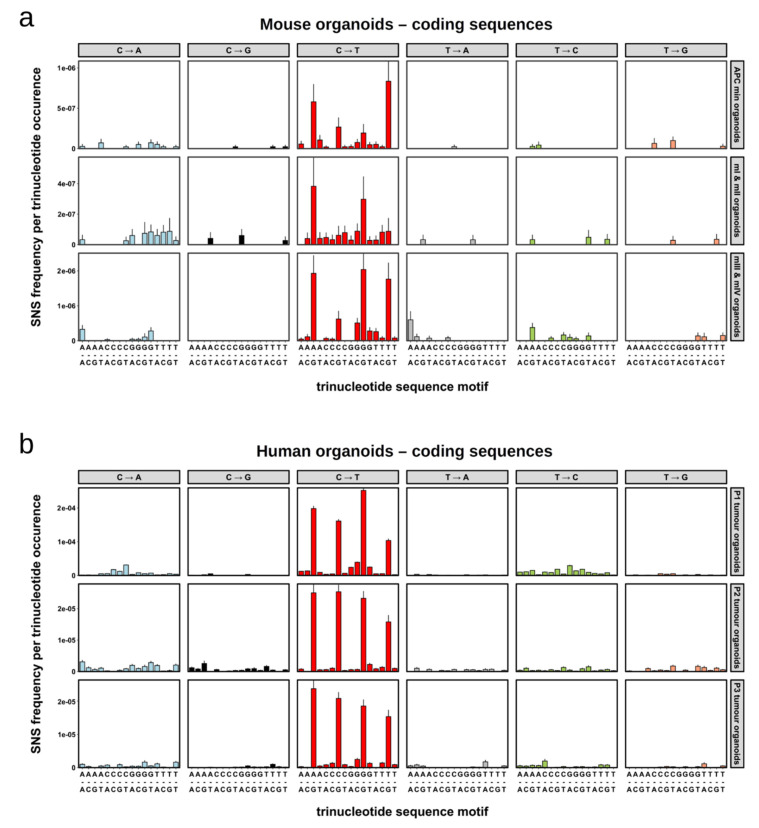
Mutational signatures of somatic SNSs in tumour organoids. (**a**) Normalised signatures of protein-coding SNSs identified in the tumour organoids from mice I-IV of this study and from *Apc^min/+^* mice. (**b**) Normalised signatures of protein-coding SNSs identified in the tumour organoids from three CRC patients. The SNS frequencies were normalised according to the prevalence of the respective nucleotide triplets in the protein-coding sequences of the mouse (**a**) and human (**b**) reference genomes.

SNSs in human cancers often target nucleotides in specific sequence contexts, which are referred to as mutational signatures. Signature 1, which is the most prevalent mutational signature in human cancers, describes the substitution of cytosines by thymines in the context of NpCpG motifs [32,41]. This signature, although present in most cancers, exhibits some tissue specificity and is particularly prevalent in precancerous lesions (adenomas) and cancers of the colon [29]. We had previously observed this signature in organoids derived from intestinal adenomas of *Apc^min/+^* mice [47]. In the current study, signature 1 was again the most prevalent signature (Figure S7a). The high prevalence of signature 1 became even more evident when the number of substitutions was normalised by the frequency of the respective triplets in the genome (Figure 4a), since the NpCpG triplet is quite underrepresented in the mouse genome (Table S1).

Interestingly, we also observed evidence for the presence of SNSs conforming to signature 17 in the mouse organoids of our current study and of our previous study of *Apc^min/+^* mice (Figure 4a and Figure S7a). Signature 17 is characterised by an elevated number of T to G and T to C substitutions in the context of CpTpT trinucleotides. Its origin is unknown, but it is particularly present in oesophageal, stomach and colon human cancers [32,60,61,62].

To determine whether the mutational profiles observed in the mouse organoids were similar to those present in human cancers, we reanalysed the published sequencing data of 36 human organoids derived from the tumours and normal tissues of three colorectal cancer patients [48]. The SNSs within the protein-coding sequences revealed a strong signature 1 profile and a weak signature 17 profile, similar to what we observed in the mouse organoids (compare Figure 4b and Figure S7b to Figure 4a and Figure S7a, respectively).

Since the human organoids were subjected to whole-genome sequencing, we were able to examine more thoroughly their mutational signature profile. At the genome-wide level, signature 1 was by far the most prevalent signature, followed by signature 17 in patients 1 and 2 (Figure S8a). Further analysis of the SNSs conforming to these signatures revealed a strong dependence on replication timing with more SNSs being present in late S than in early S replication regions (Figure S8b). Within each replication timing domain, the frequency of SNSs was similar in the protein-coding, intronic, and intergenic regions; this was true for both signatures 1 and 17 (Figure S8c). Finally, we note that the SNSs conforming to signature 1 and the C to T transitions in non-CpG contexts were the only SNS types present in organoids derived from non-transformed cells (Figure S8a).

### 2.5. Copy Number Alterations

To probe for copy number alterations (CNAs) in the mouse tumour organoids, we compared the number of sequencing reads across the genome to the number of normal tissue reads from the same mouse. For this type of analysis, whole-genome sequencing data are superior to exome sequencing data; yet the high read coverage of our data allowed us to identify copy number changes with a high degree of certainty (Figure S9).

**Figure 5:**
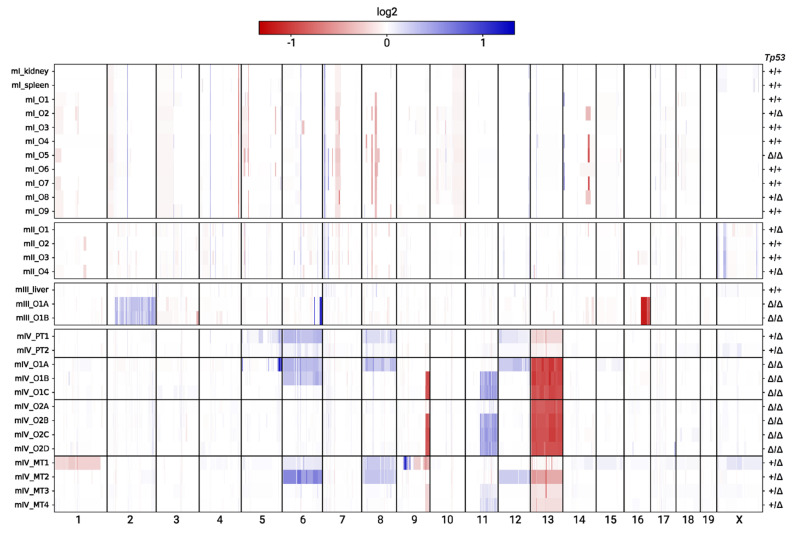
Profiles of copy number alterations (CNAs) in the tumour organoids and tissue biopsies from mice I-IV of this study. Red and blue colours correspond to deletions and duplications, respectively, and colour saturation indicates the ratio of the number of reads in the organoid or biopsy compared to the normal reference tissue. The allele status of *Tp53* is indicated on the right side of the panel. The primary and metastatic tumours of mouse IV were homozygous for *Tp53* deletion. However, the signal was diluted due to contamination by normal cells, and hence, these cases are indicated as +/Δ.

For the organoids from mice I and II, the analysis did not reveal any convincing CNAs (Figure 5). Small genomic regions with different ratios of the number of sequencing reads in the organoid and reference bulk normal tissue were observed, but similar differences were observed when comparing the number of reads between different normal tissues. Moreover, some of these differences were even shared between the organoids of mice I and II, which strongly indicates that they were noise (Figure 5).

In contrast to mice I and II, several CNAs were evident in the organoids from mice III and IV. The two organoids from mouse III shared CNAs on chromosomes 2, 6, and 16, indicating that they were related (Figure 5). These two organoids also shared 14 SNSs (Figure 2c). The seven organoids from mouse IV all shared loss of one copy of chr 13; five of the seven organoids shared CNAs on chromosomes 9 and 11; and two of the seven organoids shared a duplication of chr 6. Finally, organoid O1A had private CNAs on chromosomes 5, 8, and 12 (Figure 5). Many of the CNAs observed in the organoids from mouse IV were also evident in the primary and metastatic tumour biopsies. Interestingly, metastatic lesion 1 had several CNAs that were not present in any of the organoids or primary tumour samples; these CNAs included amplifications in chromosomes 9 and X and deletions in chromosomes 1 and 9 (Figure 5).

### 2.6. Evolution of Tumour Clones in Mouse IV

The availability of SNS and CNA data from several organoids and from primary and metastatic tumour samples of mouse IV provided an opportunity to establish an order in which these mutations were acquired during tumour evolution.

First, we plotted the allele frequencies of all the SNSs identified in the organoids and tumours of mouse IV (Figure S10). In the organoids, most allele frequencies were close to 50%, consistent with one allele being mutated in a diploid region of the genome. For a few SNSs, the allele frequencies deviated significantly from 50%, but all these SNSs were located within genomic regions affected by CNAs. In the primary tumours, the SNS allele frequencies ranged between less than 5% to about 30%: the four SNSs that were identified in all organoids had allele frequencies of about 30%; whereas the allele frequencies of the remaining SNSs were lower with the interesting exception of the SNSs that targeted the *Ociad2* and *Vmn1r119* genes, which had allele frequencies close to 30%, even though they were identified in only 3 out of the 7 organoids (Figure S10). In the metastatic lesions, all SNSs had similar allele frequencies, consistent with each metastasis having been seeded by a single cell or by a microcolony of genetically identical cells (Figure S10). Accordingly, we included the metastatic lesions in the phylogenetic tree. We note that metastatic lesions 3 and 4 had exactly the same SNSs and CNAs (Figure 2c and Figure 5).

**Figure 6:**
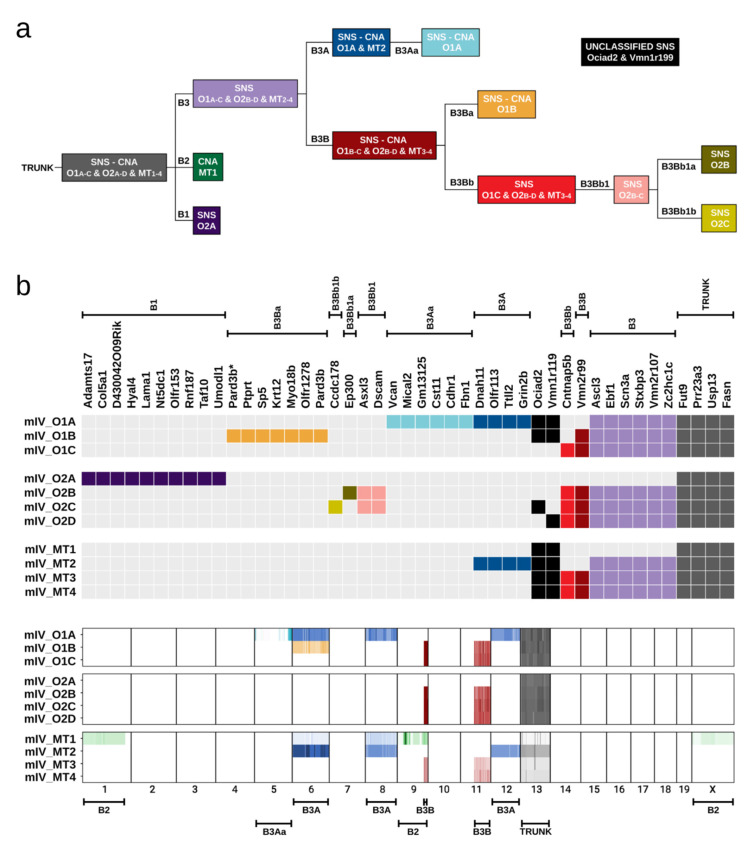
Phylogenetic analysis of tumour progression in mouse IV. (**a**) Phylogenetic tree illustrating the temporal order of acquisition of the SNSs and CNAs. The branches (B) of the tree are numbered. (**b**) Colour-coding of the SNSs and CNAs according to the phylogenetic analysis. The SNSs and CNAs belonging to the same branch of the phylogenetic tree have the same colour.

To plot the phylogenetic tree, we started with the four SNSs (SNS-TRUNK) and the deletion of one copy of chr 13 (CNA-TRUNK) that were present in all organoids and metastatic lesions (Figure 6). Three branches could be projected from the trunk of the phylogenetic tree. The first branch (B1) was formed by O2A and contained several private mutations (SNS-B1); the second branch (B2) was formed by MT1 and contained private CNAs affecting chromosomes 1, 9, and X (CNA-B2), as well as duplications of chr 6 and chr 8; the third branch (B3) was formed by all other samples and contained a group of six SNSs (SNS-B3). In regard to branch B2, we note that the duplications of chr 6 and chr 8 were also present in the O1A and MT2 samples, raising the possibility that the metastatic lesion MT1 might not be monoclonal. Therefore, we attributed only the CNAs affecting chromosomes 1, 9, and X to branch B2 (Figure 6b).

From branch B3, two branches originated; the first branch (B3A) encompassed O1A and MT2 and was characterised by the presence of four SNSs (SNS-B3A), and duplications of chromosomes 6, 8, and 12 (CNA-B3A). From this first branch, a sub-branch emerged containing O1A and characterised by a group of six SNSs (SNS-B3Aa) and amplification events in chr 5 (CNA-B3Aa). The second branch (B3B) emerging from branch B3, was characterized by a SNS targeting *Vnn2r99* (SNS-B3B), an amplification of part of chr 11 and a deletion of part of chr 9 (CNA-B3B). In turn, two branches arose from branch B3B: branch B3Ba, which was formed by O1B and was characterised by 7 private SNSs (SNS-B3Ba) and duplication of chr 6 (CNA-B3Ba; we consider this to be an independent event from the duplication of chr 6 observed in O1A and MT2) and branch B3Bb, which was formed by O1C, O2B, O2C, O2D, and MT3/MT4 and was characterised by a SNS targeting the *Cntnap5b* gene (SNS-B3Bb). From branch B3Bb, a branch (B3Bb1) containing O2B and O2C emerged; in turn, this branch gave rise to two branches characterised by SNS-B3Bb1a and SNS-B3Bb1b, respectively (Figure 6).

The phylogenetic tree encompassed all the SNSs and CNAs with the notable exception of the SNSs targeting the *Ociad2* and *Vmn1r119* genes (Figure 6), as these SNSs could not be incorporated in a way that made sense to us. What is evident is that tumour development was associated with the parallel emergence of SNSs and CNAs.

## 3. Discussion

Genomic instability is considered a major culprit of tumour development and emergence of resistance to therapy. While the presence of genomic instability in cancer was recognised at the beginning of the previous century, the advent of massive parallel sequencing has significantly advanced our understanding of the mechanisms leading to this cancer hallmark [26].

Our study focused on two major types of genomic instability: chromosomal instability and instability at the level of SNSs. The key question that we wanted to address was the extent to which the *Apc*, *Kras* and *Tp53* genes, which are frequently implicated in colon cancer development, contribute to the types of genomic instability mentioned above. As a model system, we used tumour-prone mice that express mutant *Kras* and that inactivate *Apc,* and *Tp53*. Single cell-derived organoids were examined to allow us to obtain a better understanding of cancer development in the face of tumour heterogeneity. We compared the current results to the results generated from our previous study, in which we performed exome sequencing of organoids derived from precancerous lesions and matching normal intestinal epithelium of *Apc^min/+^* mice [47].

The analysis of the data revealed a rather simple picture. The rate of accumulation of SNSs was higher in the tumour-derived organoids than in the organoids derived from the normal epithelium, but unaffected by the number of targeted cancer-driver genes. Thus, the organoids with mutant *Apc* accumulated SNSs with the same rate as the organoids with mutant *Apc* and mutant *Kras* and even the organoids with mutant *Apc*, mutant *Kras,* and mutant *Tp53*. In contrast, CNAs were present almost exclusively in the organoids harbouring mutant *Tp53*. Other studies had previously linked *Tp53* mutations to the induction of CNAs [49,63,64]. However, the observation that the rate of accumulation of SNSs was independent of the number of mutant cancer-driver genes was unexpected. We note that it remains to be determined if this is a feature of colon cancer or is more general.

As mentioned above, SNSs can exhibit specific patterns that are referred to as mutational signatures [30,32,65]. Signature 1 is the most dominant signature and is characterised by the presence of C to T substitutions in a CpG context; this signature arises from spontaneous deamination of methylated cytosines to thymines [29,42,65]. It has been proposed that signature 1 SNSs accumulate at a constant rate in both normal and transformed cells [43,44,45]. Thus, their number would reflect the age of the organism. However, in our previous study of organoids derived from *Apc^min/+^* mice, the tumour-derived organoids had a significantly higher number of CpG to TpG transitions than the organoids derived from non-transformed cells [47]. Similarly, an analysis of the sequencing data of organoids derived from three human colon cancer patients revealed more signature 1 SNSs in the tumour-derived organoids than the organoids derived from normal tissue [48] and Figure S8. Nevertheless, signature 1 SNSs were present to a significant degree in the normal tissue-derived organoids. In contrast, SNSs that do not conform to signature 1, were present almost exclusively in the tumour-derived organoids (Figure S8). We conclude that the mutagenic processes leading to signature 1 operate also in normal cells, albeit at a lower level than in tumour cells, whereas the mutagenic processes that lead to the other signatures are highly tumour-specific. Interestingly, all SNSs showed a dependence on replication timing, with late S replicating regions being significantly more prone to mutagenesis than the early S replicating regions. This dependency may explain why there is a higher density of protein-coding sequences in the early S replicating regions of the genome.

The sequencing of single cell-derived organoids from the same tumour makes it possible to construct a phylogenetic tree marking tumour development. In our study, this was possible for mouse IV. Our analysis revealed a phylogenetic tree characterised by the parallel emergence of SNSs and CNAs. In addition, analysis of metastatic lesions demonstrated that apart from MT1, the rest were derived from a single cell or genetically identical cells. In contrast, sequencing of the primary tumour tissue revealed a spectrum of mutations typical of a heterogeneous population of cancer cells.

## 4. Materials and Methods

### 4.1. Mice

Three males (mI,mII,mIV) and one female(mIII) mice were kept on a 12 h light/dark cycle in individually ventilated cages. The *Apc^lox/lox^* mice [19,66], *LSL-Kras^G12D^* mice [67] and Tp53*^lox/lox^* mice [68] were crossed to *Cdx2^CreERT2^* mice (The Jackson Laboratory, Charles River, L’Arbresle, France) [69] to obtain *Apc^lox/lox^*; *LSL-Kras^G12D^*; Tp53*^lox/lox^*; *Cdx2^CreERT2^* (AKP-*Cdx2^CreERT2^*) animals (Figure 1a,b). All experiments were authorised by the Canton of Vaud (license VD3396) and were performed according to accepted guidelines for animal handling.

### 4.2. Histopathology of Tumour and Normal Caecum Tissues

Tissues were collected, washed in PBS, fixed in 4% PFA overnight, and processed for dehydration and paraffin embedding according to standard procedures. Sections 7 μm thick were cut using a rotary microtome (Hyrax M25 V2), dried at 60 °C for 1 h and stained with hematoxylin-eosin (H&E) staining using standard protocols. Imaging was performed on an upright microscope. H&E sections were evaluated independently by two certified pathologists: Prof Vassilis Gorgoulis, Medical School, University of Athens, Greece; and Prof. Mohammad Ilyas, School of Medicine, University of Nottingham, UK.

### 4.3. Genotyping

A small amount of tissue from each mouse was used for genotyping. Confirmation of recombination events upon tamoxifen injection was assessed by genotyping the corresponding organoids that were selected for sequencing. For genotyping, the organoids from one well of a 48-well plate were lysed in 200 μL lysis buffer supplemented with 150 μg Proteinase K and then incubated overnight at 55 °C. The lysates were diluted 10 times with water and subjected to PCR amplification using GoTaq Hot Start Polymerase (M7423, Promega).

The following primers were used for genotyping: for the *Cre* allele: Cre_Fw (5′-CACCAGCCAGCTATCAACTCG-3′) and Cre_Rev (5′-TTACATTGGTCCAGCCACCAG-3′); for the *Apc^lox^* allele: Apc_Fw (5′-GTTCTGTATCATGGAAAGATAGGTGGTC-3′) and Apc_Rev1 (5′-CACTCAAAACGCTTTTGAGGGTTGATTC-3′) or Apc_Rev2 (5′-GAGTACGGGGTCTCTGTCTCAGTGAA-3′); for the *Tp53^lox^* allele: Tp53_Fw1 (5′-CACAAAAAACAGGTTAAACCCA-3′) or Tp53_Fw2 (5′-AAGGGG TATGAGGGACAAGG-3′) and Tp53_Rev (5′-GAAGACAGAAAAGGGGAGGG-3′); for the *LSL-Kras^G12D^* allele: Kras_WT_Fw (5′-TGTCTTTCCCCAGCACAGT-3′) or Kras_MUT_Fw (5′-CCATGGCTTGAGTAAGTCTGC-3′) and Kras_common_rev (5′-CTGCATAGTACGCTATACCCTGT-3’). The PCR conditions and DNA fragment sizes obtained are described in the Supplementary Information section.

### 4.4. Induction of Tumour Formation

Tamoxifen (Sigma), 3 mg/kg (or 30 mg/kg for mouse I), was administered either as a single i.p. injection with sunflower oil (mouse II) or by gavage with peanut oil (mouse I, mouse III, and mouse IV), when the mice were 10–14 weeks old. The mice were sacrificed 2 weeks (mouse I), 10 weeks (mouse II), 20 weeks (mouse III) or 25 weeks (mouse IV) after tamoxifen administration.

### 4.5. Tissue Isolation, Organoid Culture, and Expansion

Intestinal tissue (colon or caecum) was isolated from AKP- *Cdx2^CreERT2^* mice. Colonic tumours were distinct and each was dissected and treated separately. For caecum tumours, which effectively occupied nearly all the caecum space, the caecum was divided into several parts and each part was considered as a separate tumour. For each tumour, the resected tissue was cut into 2–3 mm wide cubes, that were separated by tissue also 2–3 mm wide. The tumours of mouse III, isolated 20 weeks after tamoxifen administration, were small in size and were not cut into separate pieces before processing (see Figure 1c and Figure S1b).

The tumour fragments were washed thoroughly in PBS-EDTA at 4 °C and then homogenised with a teflon pestle in 1.5 mL Eppendorf tubes. Tissue homogenates were treated with Trypsin-EDTA for 3–4 min and quickly pipetted up and down, approximately 100–200 times, using 200 µL tips to disrupt any cell aggregates. After centrifugation, the pellets were resuspended in ENR media, filtered through 70 µm cell strainers (BD Bioscience, New Jersey, NJ, USA), and single cell suspensions were mixed with cold Matrigel^®^ (Corning Glendale, AZ, USA) and plated in 96-well plates. The tissue culture media (ENR) for these organoids was based on DMEM/F12 with B27 and N2 (Life Technologies, Carlsbad, CA, USA) and contained, in addition, 10 mM HEPES, 100 units/mL penicillin and 100 µg/mL streptomycin (Life Technologies), 2 mM L-Glutamine (Life Technologies) and 1.25 µM *N*-Acetylcysteine (Sigma-Aldrich, Munich, Germany). The following growth factors were also added: 50 ng/mL murine recombinant EGF (Life Technologies), R-Spondin1-Fc, and Noggin-6xHis [70]. After the Matrigel solidified, ENR tissue culture media was added on top. The suspensions of single cells were seeded at different cell concentrations to obtain wells containing a single organoid. Selected organoids from each tumour piece were then expanded to obtain enough material for DNA sequencing. For these organoids, the medium was changed every 2 days and organoids were split every 3–4 days by mechanical dissociation. Organoids were kept in culture as short as possible to obtain the necessary amount of DNA for exome sequencing; on average, each organoid culture was split three times. Around four to six 48-well plates full of organoids were harvested and the organoid pellets were washed and frozen at −80 °C.

### 4.6. Measurement of Cell Doubling Times in Organoids

Organoids with one (Apc) or three (Apc;Kras;Tp53) mutant cancer-driver genes were trypsinized and single cells were seeded. Three days later, organoids from each condition were collected, trypsinized, and the number of cells was counted. Proliferation rate was calculated by the formula:

(1) $$\frac{duration\left(hoursafterseeding\right)*log\left(2\right)}{log\left(averagenumberofcellsperorganoidattimeofharvest\right)-log\left(averagenumberofcellsperorganoidattimeofseeding\right)}$$

### 4.7. DNA Extraction and Exome Sequencing

Genomic DNA from the organoids was extracted and fragmented by sonication. The resultant fragments (∼200 bp) were subjected to exome capture using the SureSelect Mouse All Exon Kit (Agilent Technologies, Santa Clara, CA, USA) and paired-end libraries were prepared and sequenced on an Illumina HiSeq 4000 platform.

### 4.8. Sequence Analysis

Sequencing reads were aligned on the mouse reference genome NCBI Build GRCm38/mm10 using the Burrows-Wheeler Alignment tool v.0.7.17. Bam conversion, sorting, removal of PCR duplicates, and indexing of the sequence alignment files was conducted by SAMtools v.1.9. Somatic variant calling was performed by GATK v.4.11.0 using healthy tissue from the liver or kidney of the same mouse as matching normal sample. False-positive calls were filtered out using a panel of normal samples constructed from all normal tissues of 4 mice. Variants present in common mouse dbSnp142 were also discarded. The mutational spectra of detected somatic SNSs were examined using the SomaticSignature v.2.20.0 R package for the analysis of all the 96 possible trinucleotide changes. CNA events in bam files were analysed by VarScan2 v.2.4.3 using the recommended workflow. To filter out somatic CNA events, we excluded CNAs that were present in the liver, kidney, and spleen tissues of the mice from which the organoids were prepared. Segmentation was applied by DNAcopy R package v.1.58.

### 4.9. SNS Signature Normalisation

The SNS signatures in mouse and human samples were normalised using the genomic mouse and human sequences, respectively, downloaded from the NCBI RefSeq curated dataset at the UCSC server (http://hgdownload.soe.ucsc.edu/goldenPath/ accessed on 4 October 2019). The coordinates of early-S, mid-S and late-S replicating regions of the human genome were obtained from our previous analysis of U2OS cells [71].

## 5. Conclusions

An analysis of organoids derived from a mouse model of colorectal cancer has allowed us to study the accumulation of SNSs and CNAs at the single cell level during tumour evolution. The key conclusions are that the rate of accumulation of SNSs is higher in transformed than non-transformed cells and that it is unaffected by the number of cancer-driver genes that are active in the tumour. Thus, tumours with mutant *Apc* accumulated as many SNSs as tumours with mutant *Apc*, mutant *Kras,* and mutant *Tp53*. Signature 1 SNSs are the most prevalent in our model, but are also present to a lower degree in normal cells. Moreover, late S replicating genomic regions are more prone to accumulate SNSs. In contrast to SNSs, CNAs were observed only in cells with mutant *Tp53*. So far, very few studies have been published sequencing single cell-derived tumour organoids. Nevertheless, this approach has great potential to elucidate the mutagenic processes present in cancer and, therefore, to contribute to our understanding of genomic instability.

## Data Availability

The FASTQ files of mouse exome sequencing have been deposited at the NCBI SRA database under the BioProject ID PRJNA675998.

## Supplementary Materials

The following are available online at https://www.mdpi.com/2072-6694/13/6/1267/s1, Figure S1. Mouse breeding and preparation of organoids, Figure S2. Mouse genotyping by PCR, Figure S3. Whole exome sequencing read depth of Apc genomic locus for mouse I (a), mouse II (b), mouse III (c) and mouse IV(d), Figure S4. Whole exome sequencing read depth of Kras genomic locus for mouse I (a), mouse II (b), mouse III (c) and mouse IV(d), Figure S5. Kras exon2 genotype detected by exome sequencing of mouse samples, Figure S6. Whole exome sequencing read depth of Tp53 genomic locus for mouse I (a), mouse II (b), mouse III (c) and mouse IV(d), Figure S7. Mutational signatures of somatic SNSs in tumour organoids, Figure S8. Mutational signatures of somatic SNSs in human normal and tumour cell-derived organoids as a function of replication timing and gene annotation, Figure S9. Examples of detected copy number alterations in mouse tumour organoids, Figure S10. Allele frequencies of identified somatic SNSs, Figure S11. Original Images for Figure S2, Table S1 and Data S1 attached as excel files.
